# Feasibility of a Peer Support Intervention for Family Caregivers of Black Older Adults Living with Dementia

**DOI:** 10.1093/geroni/igaf122.3933

**Published:** 2025-12-31

**Authors:** Karen Moss, Abraham Brody, Karen Bullock, Alai Tan, Kathy Wright, Mary Beth Happ

**Affiliations:** The Ohio State University, Blacklick, Ohio, United States; NYU Rory Meyers College of Nursing, New York, New York, United States; Boston College, Chestnut Hill, Massachusetts, United States; The Ohio State University, Columbus, Ohio, United States; The Ohio State University, Powell, Ohio, United States; Ohio State University, Columbus, Ohio, United States

## Abstract

Black family caregivers of older adults living with dementia are at high risk for negative impacts of caregiving, including poor overall health. Culturally responsive evidenced-based caregiving support interventions are lacking and may help to meet the unmet needs of Black family caregivers of people living with dementia. The purpose of this study was to test the feasibility and acceptability of the Peer Support for Family Caregivers of Black Older Adults Living with Dementia (Pair 2 Care) Intervention. Pair 2 Care is a 6-month, flexible, virtual peer support intervention co-designed with Black current family caregivers, former family caregivers (care recipient is deceased), community leaders, and healthcare providers. This intervention leverages the experiences of trained former caregivers as mentors to current caregiver mentees. A total of 15 current caregiver mentees were mentored by 11 trained former caregivers; all were female and mostly daughters who were caring or cared for a parent living with dementia. Pair 2 Care was found to be feasible and acceptable. All participants were enrolled, mentors were trained, and mentees and mentors were paired within 10 weeks. Former caregiver mentors were retained at 90% and mentees at 93%. Both mentors and mentees rated their overall Pair 2 Care experience as very high (9/10). Qualitative feedback was overwhelmingly positive, with several participants requesting to continue the program. Pair 2 Care may provide an innovative approach to improving family caregiver health outcomes. This award-winning intervention is poised to become part of existing innovations in aging that promotes health equity.

